# S100A9^+^ MDSC and TAM-mediated EGFR-TKI resistance in lung adenocarcinoma: the role of *RELB*

**DOI:** 10.18632/oncotarget.24146

**Published:** 2018-01-10

**Authors:** Po-Hao Feng, Chih-Teng Yu, Kuan-Yuan Chen, Ching-Shan Luo, Shen Ming Wu, Chien-Ying Liu, Lu Wei Kuo, Yao-Fei Chan, Tzu-Tao Chen, Chih-Cheng Chang, Chun-Nin Lee, Hsiao-Chi Chuang, Chiou-Feng Lin, Chia-Li Han, Wei-Hwa Lee, Kang-Yun Lee

**Affiliations:** ^1^Division of Pulmonary Medicine, Department of Internal Medicine, Shuang Ho Hospital, Taipei Medical University, Taipei, Taiwan; ^2^Division of Thoracic Medicine, Department of Internal Medicine, School of Medicine, College of Medicine, Taipei Medical University, Taipei, Taiwan; ^3^Division of Pulmonary Medicine, Department of Internal Medicine, Chang Gung Medical Foundation Linko Branch, Taoyuan, Taiwan; ^4^Graduate Institute of Clinical Medicine, College of Medicine, Taipei Medical University, Taipei, Taiwan; ^5^School of Respiratory Therapy, College of Medicine, Taipei Medical University, Taipei, Taiwan; ^6^Department of Microbiology and Immunology, School of Medicine, College of Medicine, Taipei Medical University, Taipei, Taiwan; ^7^Master Program for Clinical Pharmacogenomics and Pharmacoproteomics, College of Pharmacy, Taipei Medical University, Taipei, Taiwan; ^8^Department of Pathology, Shuang Ho Hospital, Taipei Medical University, Taipei, Taiwan

**Keywords:** lung cancer, myeloid derived suppressor cells, epidermal growth factor receptor, macrophages, NF-kappa B

## Abstract

**Background:**

Monocytic myeloid-derived suppressor cells (MDSCs), particularly the S100A9+ subset, has been shown initial clinical relevance. However, its role in EGFR-mutated lung adenocarcinoma, especially to EGFR-tyrosine kinase inhibitor (EGFR-TKI) is not clear. In a clinical setting of EGFR mutated lung adenocarcinoma, a role of the MDSC apart from T cell suppression was also investigated.

**Results:**

Blood monocytic S100A9^+^ MDSC counts were higher in lung cancer patients than healthy donors, and were associated with poor treatment response and shorter progression-free survival (PFS). S100A9^+^ MDSCs in PBMC were well correlated to tumor infiltrating CD68^+^ and S100A9^+^ cells, suggesting an origin of TAMs. Patient’s MDMs, mostly from S100A9^+^ MDSC, similar to primary alveolar macrophages from patients, both expressed S100A9 and CD206, attenuated EGFR-TKI cytotoxicity. Microarray analysis identified up-regulation of the *RELB* signaling genes, confirmed by Western blotting and functionally by *RELB* knockdown.

**Conclusions:**

In conclusion, blood S100A9^+^ MDSC is a predictor of poor treatment response to EGFR-TKI, possibly via its derived TAMs through activation of the non-canonical NF-κB *RELB* pathway.

**Methods:**

Patients with activating EGFR mutation lung adenocarcinoma receiving first line EGFR TKIs were prospectively enrolled. Peripheral blood mononuclear cells (PBMCs) were collected for MDSCs analysis and for monocyte-derived macrophages (MDMs) and stored tissue for TAM analysis by IHC. A transwell co-culture system of MDMs/macrophages and H827 cells was used to detect the effect of macrophages on H827 and microarray analysis to explore the underlying molecular mechanisms, functionally confirmed by RNA interference.

## INTRODUCTION

Lung adenocarcinoma patients with activating mutation of epidermal growth factor receptor (EGFR) had remarkable response to EGFR tyrosine kinase inhibitors (EGFR-TKIs) and better prognosis. [1, 2] EGFR-TKIs usually had 50∼70% objective response rate and 9∼11 months progress free survival (PFS), but some patient still had poor response and shorter PFS. T790M mutation and other bypass activation pathway have been extensively studied, but tumor microenvironment-mediated drug resistance is less understood. Interactions between cancer cells and tumor infiltrating cells, majorly being cancer-associated fibroblasts (CAFs), myeloid-derived suppressor cells (MDSCs) and tumor-associated macrophages (TAMs), can influence cancer progression, metastasis and even resistance to treatment. [3, 4].

MDSCs are recruited from bone marrow to peripheral blood [5], lymphoid organs and tumors by tumor-derived factors [6], where they induce T cell proliferation arrest, decrease T cell activation, inhibit NK cells, and induce regulatory T cell (Treg). [7] We have previously identified a subset of MDSCs - CD14^+^S100A9^+^ monocytic MDSCs (hereafter as S100A9^+^ MDSC) with significantly clinical relevance. In addition to confer T cell suppression, MDSCs is associated with poor response to chemotherapy in patients with non small cell lung cancer (NSCLC) [8]. It is not clear how this resistance is mediated. Also intriguingly, whether the MDSC is also linked to resistance to EGFR-TKIs in EGFR mutated lung adenocarcinoma.

Mononuclear MDSCs can further mature into TAMs in tumor microenvironment in mice model, [9] but the association of S100A9^+^ monocytic MDSCs and TAMs in lung cancer patient is not clear. TAMs promote tumor growth, angiogenesis, tumor invasion and tumor metastasis [10, 11]. In tumor bearing mice models, TAMs also induce chemotherapy resistance through various mechanisms, such as secretion of “chemoprotective” factors including lysosomal enzyme, cathepsins B and S. [12, 13] TAMs tends to be alternative activated, or M2, macrophages, characterized by high production of anti-inflammatory cytokine, IL-10, and up-regulation of cell-surface scavenger receptors, such as mannose receptor (CD206) and hemoglobin scavenger receptor (CD163). [14] High frequency of TAMs in the tumor stroma is usually associated with poor prognosis in lung cancer [15, 16]. In addition, we have provided evidence that total TAMs were correlated to treatment response of EGFR-TKI in EGFR unselected advanced lung cancer patients ^16^. Whether this is also true for patients with EGFR-mutated lung cancer is not clear, neither is the underlying molecular mechanisms.

In this report, we correlated S100A9^+^ MDSCs and TAMs to EGFR-TKI treatment response in patients with EGFR mutated lung adenocarcinoma and addressed the question as to whether this MDSC is a source of TAMs. The molecular mechanism whereby TAMs mediate EGFR-TKI resistance was also explored.

## RESULTS

### Baseline characteristics and clinical response

Totally fifty-eight patients with stage IV EGFR-mutated lung adenocarcinoma were enrolled into this study. Baseline characteristics were summarized in Table 1. The majority of patients had L858R mutation (56.9%) and were treated with Gefitinib (79.2%). Most patients had objective response to EGFR-TKI (objective response rate 79.3%) with the median PFS of 10.2 months (95% CI, 8.8–11.5 mon) and the median OS 23.4 months (95% CI, 18.6–28.3 mon).

**Table 1:** Baseline Characteristics

Characteristics	*N* (%), *n* = 58
Gender, No. (%)	
Male	29 (50)
Female	29 (50)
Mean Age, year (range)	65.9 (42–83)
ECOG^a^ Performance status, No. (%)	
0 or 1	29 (50)
≥ 2	29 (50)
Smoking, No. (%)	
Never smoker	47 (81)
Smoker	11 (19)
Body weight loss, No. (%)	
Yes	10 (17.2)
No	48 (82.8)
EGFR mutation status	
19 Del	25 (43.1)
L858R	33 (56.9)
EGFR TKI	
Gefitinib	40 (70.2)
Erlotinib	18 (29.8)
Clinical Response, No. (%)	
CR^b^	5 (8.6)
PR^c^	41 (70.6)
SD^d^	10 (17.2)
PD	2 (3.4)
Disease Free Survival, Median, (95% CI)	10.2 (8.8–11.5)
Overall Survival, Median, (95% CI)	23.4 (18.6–28.3)

### The clinical relevance of S100A9^+^ MDSC in patients with EGFR mutated lung adenocarcinoma

**Figure 1:**
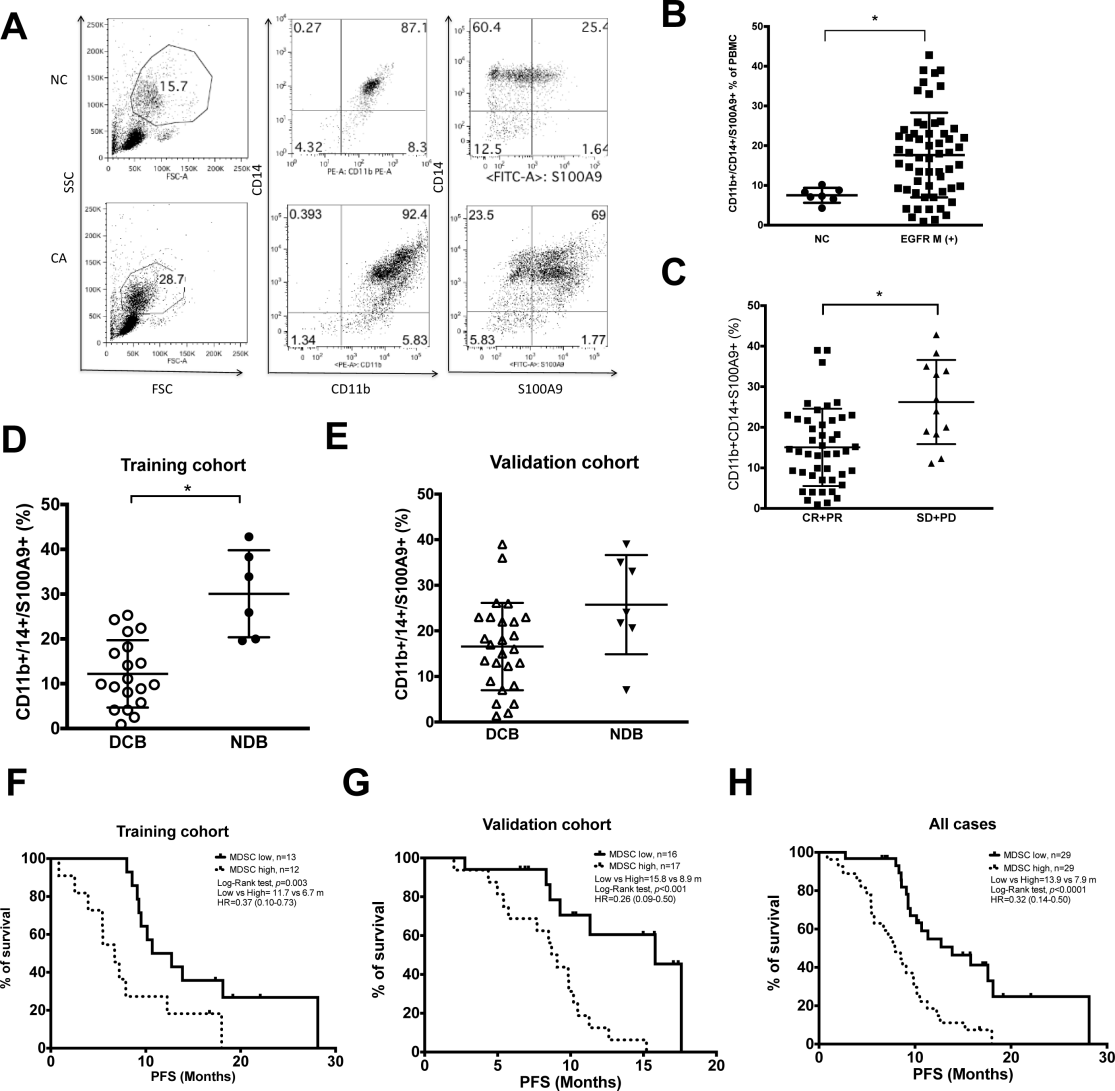
Clinical relevance of CD11b^+^CD14^+^S100A9^+^ MDSCs in lung adenocarcinoma harboring activating EGFR mutation (**A**) Representative dot plots of PBMC of NSCLC patients (CA) and normal health donors (NC). Non-lymphocyte mononuclear cell was gated, and CD11b^+^CD14^+^ S100A9^+^ cells were analyzed by flow cytometry. (**B**) Ratio of CD11b^+^CD14^+^ S100A9^+^ cells in PBMC of health donors (NC, n =7) and NSCLC patients (CA, *n* = 58). (**C**) Clinical response of EGFR-TKI treatment and peripheral blood CD11b^+^CD14^+^ S100A9^+^ MDSCs, CR+PR, *n* = 46; SD+ PD, *n* = 12. (**D**) CD11b^+^CD14^+^ S100A9^+^ difference between DCB and NCB groups in training cohort. (DCB, *n* = 19; NCB, *n* = 6, *p* = 0.0007) (**E**) CD11b^+^CD14^+^ S100A9^+^ difference between DCB and NCB groups in validation cohort. (DCB, *n* = 26; NCB, *n* = 7, *p* = 0.06) (**F–H**) Kaplain-Meier curve of PFS according to median percentage of CD11b^+^CD14^+^S100A9^+^ in training cohort, validation cohort and all cases. Dark line: CD11b^+^CD14^+^S100A9^+^ ≤18.9% in PBMC, dashed line: CD11b^+^CD14^+^S100A9^+^ > 18.9%. All data express as mean ± SD, ^*^ *p* < 0.05; CR: complete response, PR: partial response, SD: stable disease, PD: progress disease, DCB: durable clinical benefit, NDB: non-durable benefit.

The percentage of blood S100A9^+^ MDSC in the EGFR-mutated lung adenocarcinoma patients (17.7 ± 10.6, *n* = 58) was higher than that of healthy donors (7.5 ± 1.8, *n* = 7, *p* = 0.008). (Figure 1A and 1B) Patients with worse response to EGFR-TKIs had higher levels of S100A9^+^ MDSC compared with those with better response (Figure 1C, CR+PR vs SD+PD: 15.1 ± 9.5 vs. 26.2 ± 10.4, *p* = 0.002). To validate the clinical value of MDSC, we use the twenty-five patients recruited from Linko Chang Gung Memorial Hospital as the training group, and the rest 33 patients recruited from Taipei Medical University-Shuang Ho Hospital as the validation group. Patients in both groups were further divided into two sub-groups-those with durable clinical benefit (DCB) and non-durable benefit (NDB) as described. In the training group, levels of S100A9^+^ MDSC were higher in patients with NDB compared with those with DCB group (Figure 1D, 12.2 ± 7.5 vs. 30.1 ± 9.7, *n* = 19 vs. 6, *p* = 0.0007). A similar trend, although not significant, was also seen in the validation group. (Figure 1E, DCB vs NDB: 16.6 ± 9.6 vs 25.7 ± 10.9, *p* = 0.06, *n* = 26 vs 7)

To see whether blood S100A9^+^ MDSC count could be a predictor for durable response to EGFR-TKIs, we use training group data to calculate receiver operator characteristic (ROC) curve. With an area under the ROC curve (AUC) of 90%, a cutoff value of 18.9% was chosen (sensitivity 100%, specificity 75%, Supplementary Figure 1A and 1B). Applying this to the validation group, the sensitivity and specificity for DCB was 75% and 76%, respectively. (AUC: 0.71, 95% CI: 0.45–0.96, Supplementary Figure 1C and 1D) Using this cut-off value, patients with lower blood S100A9^+^ MDSC levels were consistently associated with longer PFS in the training, the validation and the overall cohorts (Figure 1F to 1H:11.7 vs. 6.7 months, *n* = 19 vs. 6, *p* = 0.003; 15.8 vs. 8.9 months, *n* = 17 vs. 16, *p* < 0.001; 13.9 vs. 7.9 months, *n* = 31 vs. 27, *p* < 0.0001, respectively). These data suggested that low percentage of peripheral blood S100A9^+^ MDSC is a good predictor for durable response of EGFR-TKI treatment.

### Correlation of peripheral blood S100A9^+^ MDSCs and TAMs in tumor tissue

**Figure 2:**
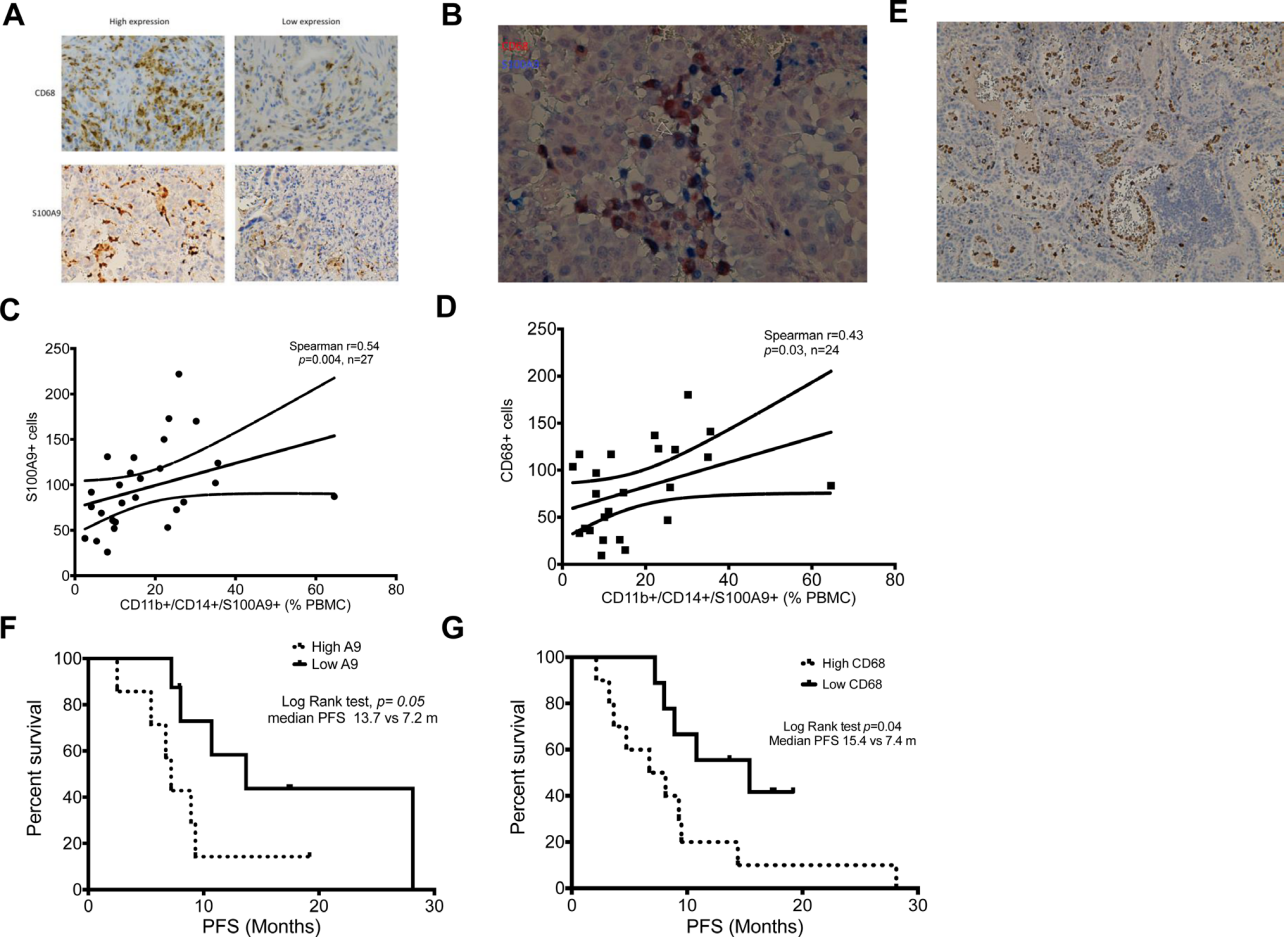
Correlation of MDSCs and TAMs between peripheral blood and tumor microenvironment (**A**). Representative immunochemistry image of CD68^+^ and S100A9^+^ cells in lung adenocarcinoma tissue. 200×. (**B**) Representative immunochemistry image of double stain of CD68 (Brown) and S100A9 (Blue) of lung adenocarcinoma. 200×. (**C**) Correlation between percentage of CD11b^+^CD14^+^S100A9^+^ MDSCs in peripheral blood and number of S100A9^+^ cells in tumor tissue. (**D**) Correlation between percentage of CD11b^+^CD14^+^S100A9^+^ MDSCs in peripheral blood and number of CD68^+^ cells in tumor tissue. (**E**) Representative immunochemistry image of S100A9^+^ cells (brown) in tumor tissue and inner lumen of blood vessels. 100× (**F**) Kaplain-Meier curve of PFS according to median number of S100A9^+^ cells in tumor tissue. Dark line: low S100A9^+^ cells, dashed line: high S100A9^+^ cells. (**G**) Kaplain-Meier curve of PFS according to median number of CD68^+^ cells in tumor tissue. Dark line: low CD68^+^ cells, dashed line: high CD68^+^ cells.

We have reported that TAMs in tumor tissue was correlated to treatment failure of first line EGFR-TKI in an *EGFR* mutation unselected cohort. [17] Evidence showed peripheral blood monocytes are one origin of recruited macrophages in human. [18] To provide clinical evidence supporting that S100A9^+^ MDSCs [19, 20], are one source of TAMs, we analyzed the tumor infiltrating CD68^+^ TAMs to compare with blood S100A9^+^ MDSCs of same patients. (Figure 2A) In tumor tissue, we were able to show CD68/S100A9 double stained TAMs, suggesting their S100A9^+^ MDSC origin. (Figure 2B) We further found that percentages of blood S100A9^+^ MDSCs were well correlated with counts of S100A9^+^ cells and CD68 TAMs in tumor tissues. (Figure 2C and 2D: S100A9^+^ MDSCs vs. S100A9^+^ cells in tumor tissue, Spearman *r* = 0.54, *p* = 0.004, *n* = 27; S100A9^+^ MDSCs vs. CD68^+^ cells, *r* = 0.43, *p* = 0.03, *n* = 24). Our IHC study also showed a plenty of S100A9-satined cells adhering to the inner surface and spreading in the vicinity of blood vessels in tumor tissues (Figure 2E). Patients with higher numbers of both S100A9^+^ and CD68^+^ cells had worse PFS. (Figure 2F and 2G: High vs. low S100A9 or CD68: 7.2 vs. 13.7 months, *p* = 0.05, *n* = 10 and 9 in S100A9 group; 7.4 vs. 15.4 months, *p* = 0.04, *n* = 7 and 8 in CD68 group, respectively). Taken together, the association of blood S100A9^+^ MDSCs with treatment response to EGFR-TKIs might be through an interaction with tumor cells by themselves and their derived macrophages.

### Phenotyping of CA-MDMs and TAMs

**Figure 3:**
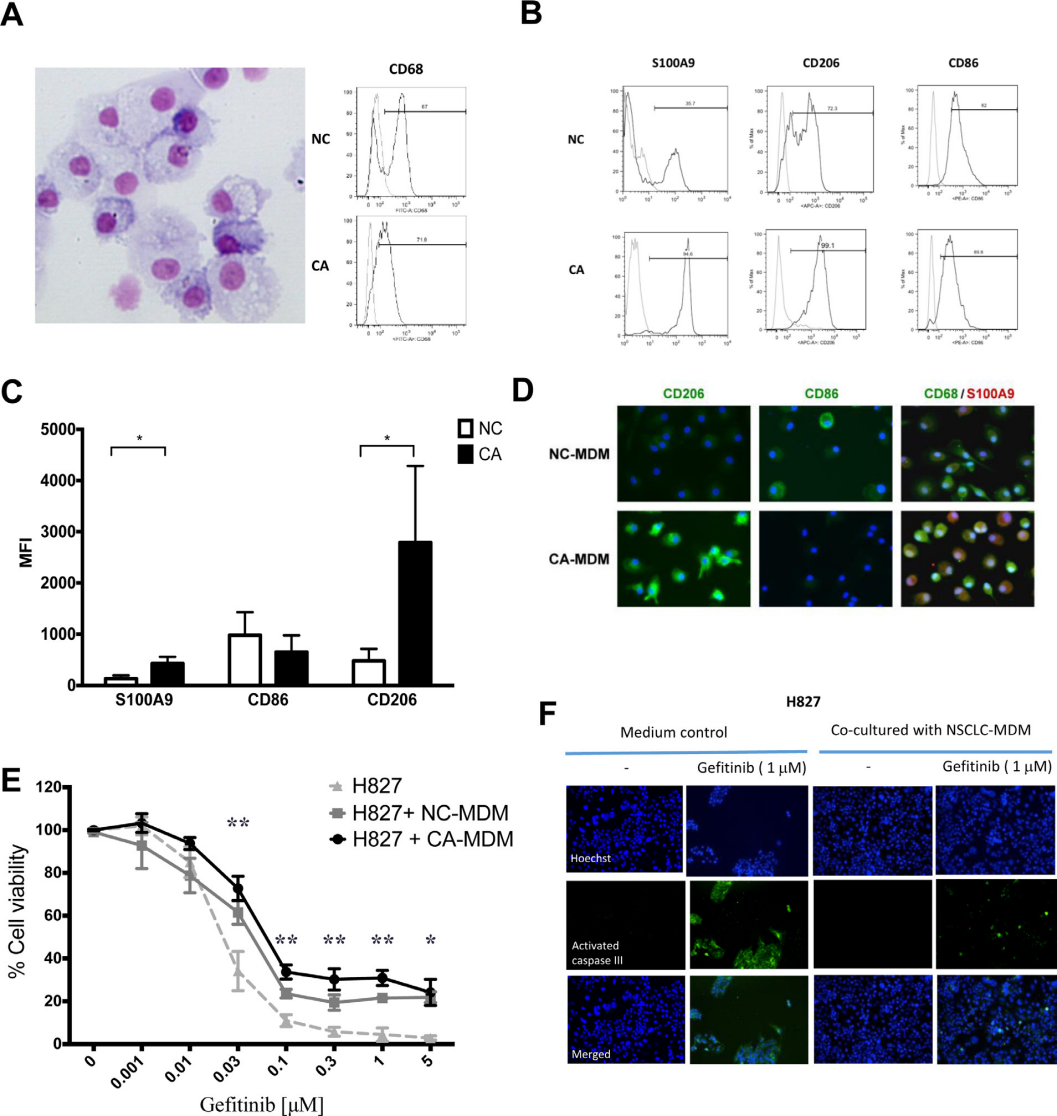
Phenotype and function of MDSCs derived macrophages (**A**) Left panel: Representative figure of monocyte-derived macrophages (MDM). 400×. Right panel: Expression of CD68 of MDM from heath donor (NC) or lung cancer patients(CA). (**B**) Representative histogram of marker of MDM from NC or CA. (**C**) Flow cytometry analysis of MDM marker from NC or CA. Data express as mean ± SD, ^*^ *p* < 0.05, *n* = 4 (**D**) Representative immunofluorescence image of MDM from NC and CA. (**E**) The MTT assay of H827 viability with transwell co-cultured with NC-MDM and CA-MDM, and treated with designated concentration of gefitinib. Data express as mean ± SEM, ^**^ *p* < 0.01, ^*^ *p* < 0.05, *n* = 3 (**F**) Apoptotic assay of H827 with transwell co-cultured with NC-MDM or CA-MDM.

To test the hypothesis that the S100A9^+^ MDSC-derived macrophages can protect cancer cells from EGFR-TKI killing, we isolated CD14^+^ monocytes from PBMCs and induce them into macrophages (monocytes-derived macrophages, MDMs). (Figure 3A) MDMs from lung cancer patients (CA-MDM), being derived from S100A9-enriched monocytes, retained higher expression of S100A9 and expressed stronger CD206, a marker for M2 macrophages. (Figure 3B and 3C, NC vs. CA, S100A9 MFI: 109 ± 64 vs. 400 ± 132, *p* < 0.05, *n* = 4; CD206 MFI: 477 ± 236 vs. 2417 ± 1498, *p* < 0.05, *n* = 4). CA-MDMs and MDMs from healthy donors (NC-MDMs) had similar expression levels of CD86, a M1 marker. The phenotyping of CA- and NC-MDMs was confirmed by immunofluorescence analysis. (Figure 3D)

**Figure 4:**
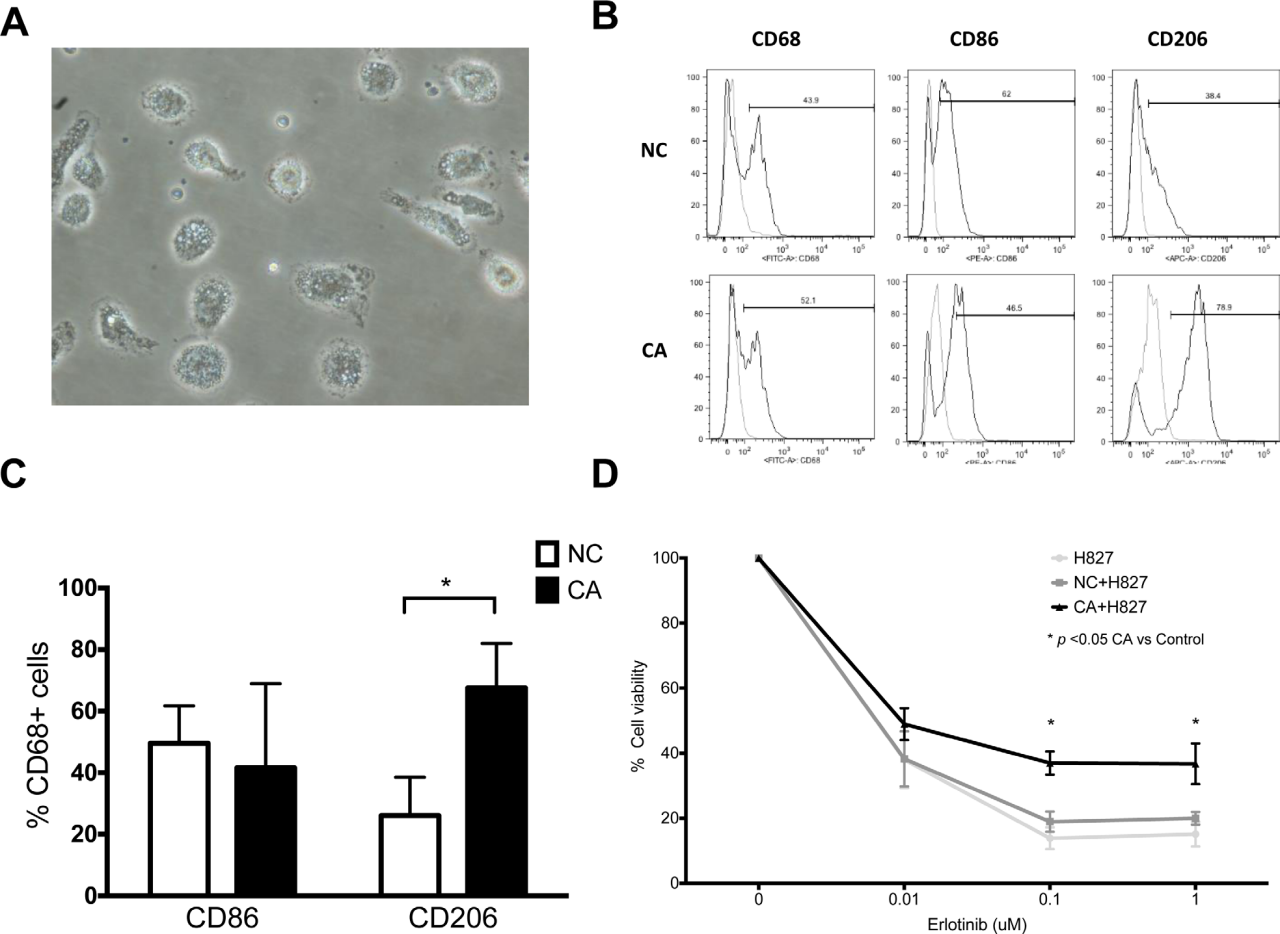
Phenotype and function of bronchoalveolar lavage macrophages (**A**) Representative microscopic image of bronchoalveolar lavage macrophages. 40×. (**B**) Representative histogram of macrophages from healthy donor (NC) and lung cancer patients (CA). (**C**) Expression of CD86 and CD206 of bronchoalveolar lavage macrophages. (**D**) MTT assay cell viability of H827 co-cultured with BALF macrophages from NC and CA. Both cells were treated with designated concentration of erlotinib. *n* = 7. Figure presented as mean ± SEM. ^*^p < 0.05.

To understand the phenotype of real macrophages in lung cancer, we also isolated macrophages from BALF. Whereas macrophages from both lung cancer patients and healthy donors expressed similar levels of CD86, the former had stronger CD206. (Figure 4B and 4C, NC vs. CA, CD86: 49.5 ± 12.1 vs. 41.6 ± 27.3, *n* = 4, *p* > 0.05; CD206: 26.1 ± 12.4 vs. 67.6 ± 14.4, *n* = 4, *p* < 0.05). It is therefore clear that CA-MDMs have a similar phenotype as lung cancer TAMs, both skewing to the M2 phenotype.

### CA-MDMs and TAMs attenuated EGFR-TKI cytotoxicity

We next performed a series of *in vitro* studies using a trans-well MDM/lung cancer cell co-culture system. EGFR-TKI sensitive NSCLC cell lines including H827, H4006 and H2935 had been tested. As the initial results were similar, H827 was used for the whole study. H827 alone was very sensitive to gefitinib; the majority of the cells were killed at the concentrations over 0.1 μM and the IC50 of gefitinib was 0.018 μM. When co-cultured with CA-MDMs, the viable H827 cells remarkably increased in the presence of gefitinib at the concentrations over 0.03 μM compared with medium controls. The viability was 72.7 ± 5.7 % vs. 34.1 ± 9.1% (*p* < 0.01), 33.6 ± 3.3% vs. 11.0 ± 2.8% (*p* < 0.01), 30.2 ± 4.9& vs. 5.7 ± 2.0% (*p* < 0.01), and 30.9 ± 3.5% vs. 4.5 ± 3.1% (*p* < 0.01), 24.1 ± 6.1%vs. 2.9 ± 1.0% (*p* < 0.05) at 0.03 μM, 0.1 μM, 0.3 μM, 1 μM and 5 μM of gefitinib, respectively. (Figure 3E) Confocal microscopy analysis of cleaved caspase III, confirmed that CA-MDMs strikingly protected H827 cells from gefitinib-induced apoptosis at 24 h. (Figure 3F, right panel) Although NC-MDMs had a trend of protection, the effect was weaker and did not reach significance. As the majoritiy of the CA-MDMs is S100A9^+^ MDSCs, our data support that S100A9^+^ MDSC-derived macrophages, but not normal MDMs, can attenuate EGFR-TKI cytotoxicity.

Although TAM-mediated drug resistance has been extensively studied in animal models, direct evidence from primary human TAMs were limited. Macrophages isolated from BALF were therefore used for the trans-well studies (Figure 4A). Protective effects of TAMs on H827 cells were also observed to the same extend as CA-MDM. (Figure 4D) Viability of H827 cells was 36.9 ± 3.5% vs. 14.1 ± 2.7% (each *n* = 6, *p* < 0.05) and 36.7 ± 6.2% vs. 15.8 ± 3.0% (each *n* = 6, *p* < 0.05) at 0.1 μM and 1 μM of erlotinib, TAM vs. medium control, respectively. By contrary, primary macrophages from healthy donors did not show significant effects on H827 cells.

### Molecular mechanisms underlying CA-MDM-mediated EGFR-TKI resistance

**Figure 5:**
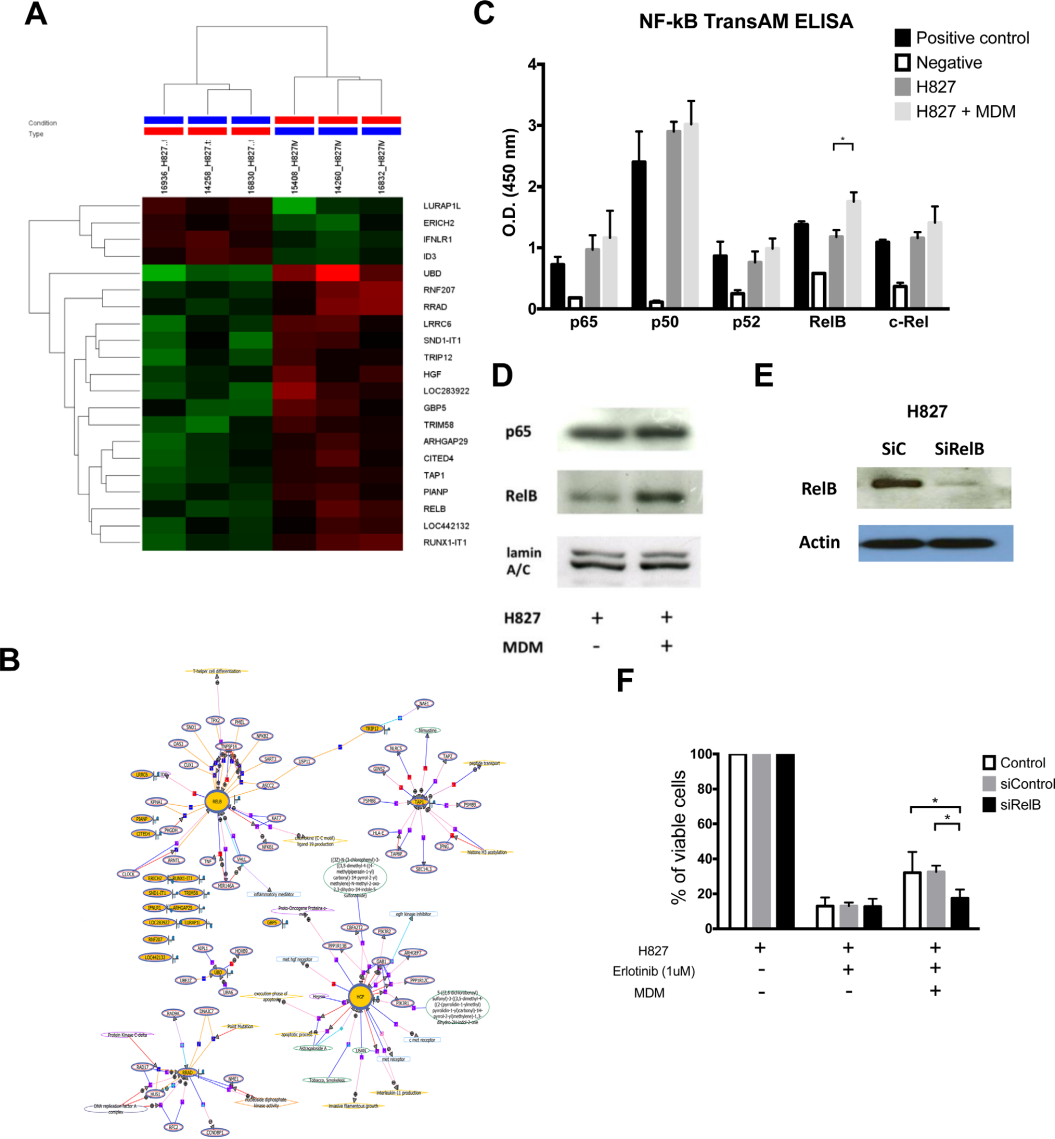
Mechanism of MDSCs/TAMs induced EGFR-TKI resistance (**A**) Hierarchical clustering of H827 with or without co-cultured with MDSCs derived macrophages. (MDM) (**B**) Network analysis result of microarray data of H827 with or without co-cultured with MDM. (**C**) NF-κB DNA binding ELISA of H827 with or without co-cultured with MDM. (**D**) Western blot of nuclear protein, p65 and RelB, of H827 cells with or without co-cultured with MDM. (**E**) Western blot of RelB knockdown of H827 cells. (**F**) Knockdown of RelB of H827 attenuated the protective effect of co-cultured with MDM.

To understand the underlying mechanisms whereby H827 cells are protected by CA-MDMs, we performed gene expression microarray analysis on the co-cultured H827 cells. Clusters of expressed genes with significant difference were illustrated in Figure 5A, showed with a heat-map. Network analysis using Natural Language Processing (NLP) algorithm showed both *RELB* and *HGF* are the centers of two gene groups (Figure 5B). To validate the array results, *RELB* expression in H827 cells was determined by a NF- **κ** B DNA binding ELISA. It was demonstrated that the DNA binding activity of *RELB*, but not the canonical pathway p65/p50, was stimulated by co-cultured CA-MDM compared with the medium controls. (Figure 5C. 1.755 ± 0.075 vs. 1.179 ± 0.056, *n* = 4, *p* = 0.02). Activation of *RELB* was also confirmed by Western blotting analysis of nuclear translocation. (Figure 5D) *RELB* knockdown (Figure 5E, SiRelB) restored the cytotoxicity of erlotinib on H827 cells in the presence of co-cultured CA-MDMs compared with no transfection controls (Control) and scramble RNA controls (SiControl) (Figure 5F, cell viability, Control vs SiRelB: 32.1 ± 11.8% vs. 17.5 ± 5.0%, each *n* = 3, *p* < 0.05; SiControl vs. SiRelB: 32.5 ± 3.5 vs. 17.5 ± 5.0, *n* = 3, *p* < 0.05) The results support that CA-MDMs could induce EGFR-TKI resistance, at least in part, through up-regulation of the non-canonical *RELB* pathway.

## DISCUSSION

In the study, we showed the clinical relevance of S100A9^+^ MDSCs and TAMs in terms of microenvironment-mediated resistance to EGFR-TKIs in the setting of lung adenocarcinoma harboring activating EGFR mutations and linked the circulating S100A9^+^ MDSCs to tumor microenvironment. Although MDSCs have been extensively studied in tumor-bearing animals, only a handful of human data are available. In tumor-bearing mice, MDSCs express CD11b and unique marker, Gr-1, but there is no Gr-1 homologue in human. In cancer patients, a number of MDSCs subtypes were reported, usually classified as three major phenotypes: monocytic, granulocytic and immature MDSCs. [21] The clinical relevance of MDSCs in human cancer is less studied, mostly focused on correlation between high levels of monocytic MDSCs and shorter OS or PFS in different cancers [21]. We previously identified a new subset of monocytic MDSC, e.g. S100A9^+^ MDSC, in patients with NSCLC [19]. This distinct MDSC was further supported by Zhao *et al.* [22] in patients with gastric cancer. S100A9^+^ MDSC is an inflammatory monocyte [19, 20], in which S100A9 is essential for accumulation of MDSC [23]. In this study, we have extended in this field that S100A9^+^ MDSCs are closely associated with treatment response and PFS in patients treated with EGFR-TKI, validated by independent cohorts.

The relation between these monocytic MDSCs and TAMs was not clear in human. Macrophages originate from blood monocytes (MDMs, monocytes-derived macrophages) or tissue-resident macrophages (TRMs) arise from yolk sac progenitors [24]. Most mice studies argued that the majority of TAMs are derived from blood monocytes, recruited to tumor site by tumor-derived factors, and phenotypically and functionally distinct from TRMs [25]. Despite of different cell surface markers, TAMs and MDSCs share some similar functions, including inducing Treg cells and upregulation of ARG1, iNOS or indoleamine 2,3-dioxygenase 1 (IDO1) and IDO2 [26, 27]. In our study, we showed that monocytic S100A9^+^ MDSCs is well correlated to tumor infiltrating TAMs, and that NSCLC MDMs retained S100A9 and phenotypically had the M2 marker CD206. Functionally, these S100A9^+^ MDSC-derived macrophages could induce EGFR-TKI resistance as the primary alveolar macrophages. Taken together, the clinical association of circulating S100A9^+^ MDSC with treatment response to EGFR-TKI is, at least in part, due to it derived macrophages effect.

Although TAMs in tumor tissue have been shown to associate with poor response of EGFR-TKIs in lung cancer patients [28], the underlying mechanisms were poorly understood. We have validated the results from network analysis, focusing on *RELB*, the alternative NF- κB pathway. Activation of *RELB* was confirmed by NF- **κ**B DNA binding ELISA and Western blotting, whilst its function role by RNA interference. Activation of *RELB* in cancer cells is supported by Dimitrakopoulos *et al.* demonstrated by IHC study of tumor tissues from patients with NSCLC. [29]. Bivona *et al.* showed that silencing of FAS or *RELA* induced regression and apoptosis of erlotinib-resistant tumor cell lines upon treatment. [30] Bivona’s conclusion was based on the observation of lung cancer cell lines alone; however our study was conducted by co-culture of lung cancer cell lines with MDSC-MDMs. Activation of RELB had multiple downstream effects, including CD4+ T cell differentiation, [31] induction of indoleamine 2,3-dioxygenase (IDO) [32] and even influence canonical pathway by WW domain–containing oxidoreductase. [33] Down-stream to RELB is important, however, it is far beyond the foucus of this article. And recent study of Ge Q.L. *et al.* reported RELB links deregulated cell cycles and apoptosis in endometrial cancer model. [34] These data also support our results.

There were several limitations in this study. First, although our results were from two independent prospective cohorts in two academic hospitals, patient number is relative small. Second, due to difficulty in tracking S100A9^+^ MDSC in the human body, we correlated tissue CD68^+^ cells (TAMs) with tissue S100A9 cells and with circulating S100A9 MDSCs to support the S100A9 MDSC origin of TAMs. Thus we did not provide direct evidence to prove this concept. To strengthen the theory, we have performed a serious of *in vitro* phenotyping study to demonstrate the similarity of TAMs and MDCS-MDM.

## CONCLUSIONS

In conclusion, circulating S100A9^+^ MDSCs is a predictor for shorter PFS in patients with EGFR mutated lung adenocarcinom. This clinical relevance is partly due to S100A9^+^ MDSC-derived macrophages in the tumor microenvironment, which attenuate the cytotoxic effect of EGFR-TKIs on otherwise sensitive cancer cells. S100A9^+^ MDSC-derived macrophages mediate resistance to EGFR-TKIs through multiple mechanisms, including the novel *RELB* alternative NF-B pathway. In addition to a useful predictive marker, S100A9^+^ MDSCs and its related pathways, such as *RELB*, are thus potential therapeutic targets to overcome drug resistance.

## MATERIALS AND METHODS

### Subjects

This study was approved by Linko Chang-Gung Memorial hospital Institutional Review Board and Taipei Medical University Joint Institutional Review Board (TMU-JIRB No. 201402046 and CGMH IRB 101-5121 A3), and informed consent was obtained from all subjects. All subjects were stage IV lung adenocarcinoma harboring activating EGFR and received first line EGFR-TKI treatment. Tumor response was evaluated using computed tomography according to the Response Evaluation Criteria in Solid Tumors (RECIST) criteria [35]. Progress free survival (PFS) was defined as start of EGFR-TKI to image documented progress of lesion and overall survival (OS) data, was recorded. Patients with PFS at least 6 months were defined as durable clinical benefit (DCB), and others were defined as non-durable benefit (NDB). [36]

### Isolation of CD14^+^ cells and differentiation of monocyte-derived macrophage (MDM)

Isolation of CD14^+^ cells and quantification of CD11b^+^CD14^+^S100A9^+^ MDSC were as previous described. [8] For differentiation into monocyte-derived macrophage (MDM), cells will be cultured for 4 days in presence of recombinant human GM-CSF (10 ng/ml).

### Bronchoalveolar lavage (BAL) purified macrophages

Under proper consent for bronchoscopy examnication, 100 ml warm normal saline was instilled into proper lung segment to acquire adequate BAL fluid (BALF), and all collected fluid was filtered through single layer of loose sterile surgical gauze and collected in polypropylene tube (Falcon, Becton Dickinson). Cell pellets was collected by centrifugation with 2500 RPM for 10 minutes, and re-suspension to about 1 × 10^6^/ml in RPMI 1640 medium, with 10% FBS, 100 IU/ml Penicillin, 100 μg/ml Streptomycin, Nystatin 100 IU/ml. 10 ml cell suspension was plated to sterile 100 mm tissue culture dish (Thermo scientific) and incubated for one hour at 37° C with 5% humidified CO2. The adherent cells was lifted by 1ml PBS containing 5 mM EDTA for 5 min for further experiment.

### *In vitro* transwell co-culture of H827 with MDM/Alveolar Macrophages and MTT assay

We use 1.2 * 10^5^ H827 cells in lower chamber of 24-well plate and co-cultured with or without 5^*^10^5^ MDM or alveolar macrophages from NSCLC patients or normal subjects in the upper chamber of transwell polycarbonate permeable dish (0.45 μM) (Corning Incorporated, MA, USA) that are of small size to prevent migration of cells. After overnight incubation at 37° C with 5% CO2, erlotinib or gefitinib was added into the bottom chamber at designated concentration. After 4 days of culture, the viability of H827 cells were determined by MTT assay (Promega, Madison, WI) according to the manufacturer’s instructions.

### Antibodies for flow cytometry

PBMCs or specific cells were stained according to the manufacturer’s recommendations. In brief, single cell suspensions were stained with fluorochrome-labeled antibodies at dilution of 1:20 at 4° C for 15 min in PBS/1% FCS (CD11b-PE, clone ICRF44, BD, Cat 555388; CD14-PE/Alexa, Clone TUK4, Serotec, Cat MCA1568P647; CD86-FITC, clone IT2.2, BioLegend, Cat 560957; CD206-FITC, clone 15-2, BioLegend Cat 17-2069-41) For intracellular staining, the cells were permeabilized by BD fluorescence-activated cell sorter permeabilizing solution (BD Biosciences, San Jose, CA, Cat 340973) and stained with or S100A9 (Abnova, clone 4G9, Cat 350704). The data from 10,000 events were analyzed with FlowJo software (TreeStar, Inc., Ashland, OR).

### Immunohistochemistry stain

Tissue sections were cut, placed onto glass slides, de-waxed in xylene and alcohol and then washed with phosphate-buffered saline twice as our previous study. Tissue sections were stained with mouse anti-human CD68 monoclonal antibody (Clone PG-M1 from Dako, Glostrup, Denmark) or rabbit anti-human S100A9 monoclonal antibody (GeneTex) separately. The five most representative high-power fields (200 magnified) per slide were manually selected using an Olympus BX50 microscope (Olympus, Southall, United Kingdom). The nuclear cells with positive CD68 or S100A9 staining were counted by ImageJ (NIH) by independent pathologist blinded to clinical outcome. Median number of positive CD68 or S100A9 cells were use to divide the high and low expression group.

### Apoptotic assay by caspase-3 immunostaining

NSCLC cell lines will be seeded into each well of double-chamber slides (Nalge Nunc International, IL) and then treated with 1 μM Gefitinib. After 24 hours, the cells will be fixed with methanol at –20° C for 10 min and then blocked with PBS containing 1%BSA for 1 hr. Apoptotic cells will be detected by the cleaved caspase-3 (Asp-175) antibody (FITC conjugated) (Cell Signaling Technology, MA); total cell numbers will be measured by Hoechst staining.

### Western blotting

Total cellular proteins will be extracted from cell pellet in lysis buffer (50 mM Tris-HCl, pH 7.4, 150 mM NaCl, 1% NP-40, 0.25% sodium deoxycholate, protease inhibitor cocktail; Boehringer Mannheim, Lewes, UK) and cytosol/nuclear protein extracted using nuclear extraction kits; 40 mg of soluble proteins from the cell lysate was re-suspended in 4X Laemmli sample buffer. Proteins will be subjected to 10% SDS-polyacrylamide gel electrophoresis and blotted onto nitrocellulose filters. RelB and p65 will be detected with the specific antibodies and an alkaline phosphatase-conjugated anti-rabbit secondary antibody (1:10000 dilution, Calbiochem, La Jolla, CA). Blots will be incubated with enhanced chemiluminescence solution (LumiGLO, Bioscience). Images will be acquired and analyzed using G:BOX (Syngene Frederick, MD).

### NF-kB DNA binding ELISA NF-kB

Activation of NF-κB family will be measured using TransAM NF-κB family kits (Active Motif, Carlsbad, CA) according to the manufacturer’s instructions. This ELISA-based method determines the DNA-binding activity of all the members of the NF-κB family (p50, p52, p65, c-Rel and RelB). Briefly, 20 μg of nuclear protein samples will be incubated for 1 hour in a 96-well plate coated with an oligonucleotide that contains a NF-κB consensus binding site (5′-GGGACTTTCC-3′), to which NF-κB contained in nuclear extracts specifically binds. After washing, one of the antibodies specific for p50, p52, p65, c-Rel or RelB (1:1000 dilution) will be added to these wells and incubated for 1 hour. Following incubation for 1 hour with a secondary HRP-conjugated antibody (1:1000 dilution), specific binding will be detected by colorimetric estimation at 450 nm with a reference wavelength of 655 nm.

### Transfection and RNA interference (RNAi)

H827 cells was transiently transfected using the Nucleofector system from Amaxa Biosystems (Invitrogen, CA), conditions optimized according the protocol provided. For nucleofaction, after centrifugation, 5 × 10^6^ cells will be suspended in 100 μl of pre-warmed Nucleofector solution containing 500 nM final concentration of siRNAs targeting RelB of Nf-κB pathway (Dharmacon) using the manufacturer’s protocols. The concentrations of plasmids and SiRNAs will be optimized. The samples will be transferred into an electroporation cuvette, and transfections will be performed with the manufacturer’s programs. After nucleofection, the cells will be immediately transferred into pre-warmed complete RPMI medium and cultured at 37° C in a humidified atmosphere of 5% CO2 for further experiments.

### Microarray analysis

0.2 μg of total RNA was amplified by a Low Input Quick-Amp Labeling kit (Agilent Technologies, USA) and labeled with Cy3 (CyDye, Agilent Technologies, USA) during the *in vitro* transcription process. 0.6 μg of Cy3-labled cRNA was fragmented to an average size of about 50–100 nucleotides by incubation with fragmentation buffer at 60° C for 30 minutes. Correspondingly fragmented labeled cRNA is then pooled and hybridized to Agilent SurePrint G3 Human GE 8 × 60K Microarray (Agilent Technologies, USA) at 65° C for 17 h. After washing and drying by nitrogen gun blowing, microarrays are scanned with an Agilent microarray scanner (Agilent Technologies, USA) at 535 nm for Cy3. Scanned images are analyzed by Feature extraction 10.5.1.1 software (Agilent Technologies, USA), an image analysis and normalization software is used to quantify signal and background intensity for each feature. Significant gene expression was set to *p*-value < 0.05 and > 1.5 fold change. The data discussed in this publication have been deposited in NCBI’s Gene Expression Omnibus (Edgar *et al.*, 2002) and are accessible through GEO Series accession number GSE79101 (https://www.ncbi.nlm.nih.gov/geo/query/acc.cgi?acc=GSE79101). GeneSpring (GX12) systemic default setting used for calculation of NLP algorithm. The *p-*value was also set to < 0.05.

### Statistics

Quantitative variables were assessed by Mann-Whitney test for continuous variables, and the categorical variances between groups were assessed by Kruskal-Wallis analysis. Comparisons among multiple groups will be done using repeated measure ANOVA. The relationships between two parameters were investigated using Spearman rank correlation test. Survival curves were estimated by the Kaplan-Meier method, whereas the log-rank test was used to compare the patient survival times per group. GraphPad Prism (version 6.0; GraphPad Software, San Diego, CA) was used for all statistical analyses, and statistical significance was defined as *P* < 0.05. The significance level was set at *p* < 0.05.

## SUPPLEMENTARY MATERIALS FIGURE



## Abbreviations

CAFs: cancer-associated fibroblasts
EGFR: epidermal growth factor receptor
EGFR-TKI: epidermal growth factor receptor-tyrosine kinase inhibitor
MDSCs: myeloid-derived suppressor cells
MDMs: monocyte derived macrophages
NSCLC: non small cell lung cancer
OS: overall survival
PFS: progress free survival
RECIST: Response Evaluation Criteria in Solid Tumors
