# Correction: S100A9+ MDSC and TAM-mediated EGFR-TKI resistance in lung adenocarcinoma: the role of RELB

**DOI:** 10.18632/oncotarget.25943

**Published:** 2018-07-31

**Authors:** Po-Hao Feng, Chih-Teng Yu, Kuan-Yuan Chen, Ching-Shan Luo, Shen Ming Wu, Chien-Ying Liu, Lu Wei Kuo, Yao-Fei Chan, Tzu-Tao Chen, Chih-Cheng Chang, Chun-Nin Lee, Hsiao-Chi Chuang, Chiou-Feng Lin, Chia-Li Han, Wei-Hwa Lee, Kang-Yun Lee

**Affiliations:** ^1^ Division of Pulmonary Medicine, Department of Internal Medicine, Shuang Ho Hospital, Taipei Medical University, Taipei, Taiwan; ^2^ Division of Thoracic Medicine, Department of Internal Medicine, School of Medicine, College of Medicine, Taipei Medical University, Taipei, Taiwan; ^3^ Division of Pulmonary Medicine, Department of Internal Medicine, Chang Gung Medical Foundation Linko Branch, Taoyuan, Taiwan; ^4^ Graduate Institute of Clinical Medicine, College of Medicine, Taipei Medical University, Taipei, Taiwan; ^5^ School of Respiratory Therapy, College of Medicine, Taipei Medical University, Taipei, Taiwan; ^6^ Department of Microbiology and Immunology, School of Medicine, College of Medicine, Taipei Medical University, Taipei, Taiwan; ^7^ Master Program for Clinical Pharmacogenomics and Pharmacoproteomics, College of Pharmacy, Taipei Medical University, Taipei, Taiwan; ^8^ Department of Pathology, Shuang Ho Hospital, Taipei Medical University, Taipei, Taiwan

**This article has been corrected:** The correct funding information is listed below:

This study was supported by National Science Council (NSC 102-2314-B-038-048) to PHF, Ministry of Science and Technology (MOST 103-2314-B-038 -056 and 104-2314-B-038-070) to PHF, and Taipei Medical University (TMU103-AE1-B31, TMU103-AE2-I01-3, 103TMU-SHH-01-2, 105TMU-SHH-02-2) to PHF.

Original article: Oncotarget. 2018; 9:7631-7643. https://doi.org/10.18632/oncotarget.24146

