# Supplementary material for: S100A9+ MDSC and TAM-mediated EGFR-TKI resistance in lung adenocarcinoma: the role of RELB
Source: Oncotarget. 2018 Jan 10;9(7):7631–43. doi: 10.18632/oncotarget.24146 (PMC5800931; doi:10.18632/oncotarget.24146)
Supplement: Supplementary file 1 [file oncotarget-09-7631-s001.pdf]

S100A9[+] MDSC and TAM-mediated EGFR-TKI resistance in lung adenocarcinoma: The role of *RELB*

SUPPLEMENTARY MATERIALS

A

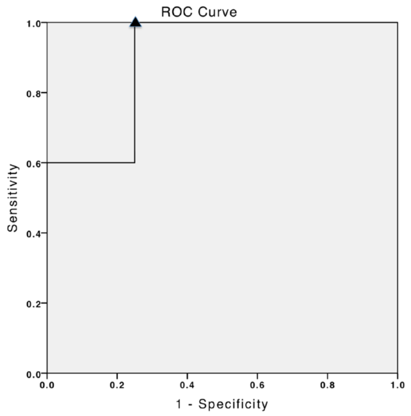

B

Coordinates of the Curve

Test Result Variable(s): MDSC_preTx

Positive if Greater Than or Equal To[a]	Sensitivity	1 – Specificity
-.1000	1.000	1.000
1.7000	1.000	.950
3.3000	1.000	.900
4.9400	1.000	.800
6.9400	1.000	.750
8.5000	1.000	.700
9.1000	1.000	.650
9.5500	1.000	.600
9.8500	1.000	.550
10.5000	1.000	.500
12.6000	1.000	.450
14.3500	1.000	.400
15.7000	1.000	.350
17.5100	1.000	.300
18.9100	1.000	.250
19.8000	.800	.250
20.8250	.600	.250
22.0250	.600	.200
23.3500	.600	.150
24.8000	.600	.100
25.6000	.600	.050
29.9000	.600	.000
36.1000	.400	.000
40.5500	.200	.000
43.8000	.000	.000

C

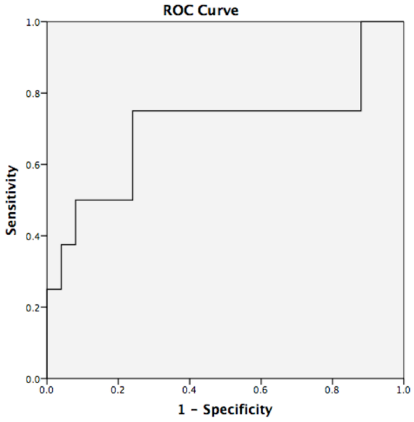

D

Coordinates of the Curve

Test Result Variable(s): MDSC_preTx

Positive if Greater Than or Equal To[a]	Sensitivity	1 – Specificity
.3700	1.000	1.000
1.5850	1.000	.960
2.6500	1.000	.920
3.5100	1.000	.880
5.0600	.875	.880
6.7000	.750	.880
7.6000	.750	.840
9.0000	.750	.800
10.9300	.750	.760
12.7800	.750	.720
13.3500	.750	.680
13.4400	.750	.600
13.4900	.750	.560
13.8500	.750	.520
14.8500	.750	.480
16.0000	.750	.440
17.1000	.750	.400
18.0000	.750	.360
18.3600	.750	.320
18.5100	.750	.280
19.5950	.750	.240
21.1400	.625	.240
21.8450	.500	.240
22.3500	.500	.200
22.8500	.500	.160
23.1500	.500	.120
23.7000	.500	.080
25.1100	.375	.080
30.9600	.375	.040
37.3950	.250	.040
38.9950	.250	.000
40.3500	.125	.000
42.7000	.000	.000

Supplementary Figure 1: (**A and B**) ROC curve and coordinates of the curve of training group. (**C and D**) ROC curve and coordinates of the curve of validation group.
